# Human CLEC9A antibodies deliver Wilms' tumor 1 (WT1) antigen to CD141^+^ dendritic cells to activate naïve and memory WT1‐specific CD8^+^ T cells

**DOI:** 10.1002/cti2.1141

**Published:** 2020-06-12

**Authors:** Frances E Pearson, Kirsteen M Tullett, Ingrid M Leal‐Rojas, Oscar L Haigh, Kelly‐Anne Masterman, Carina Walpole, John S Bridgeman, James E McLaren, Kristin Ladell, Kelly Miners, Sian Llewellyn‐Lacey, David A Price, Antje Tunger, Marc Schmitz, John J Miles, Mireille H Lahoud, Kristen J Radford

**Affiliations:** ^1^ Cancer Immunotherapies Laboratory Mater Research Institute — The University of Queensland Translational Research Institute Woolloongabba Australia 4102 Australia; ^2^ Infection and Immunity Program Monash Biomedicine Discovery Institute and Department of Biochemistry and Molecular Biology Monash University Clayton VIC Australia; ^3^ Division of Infection and Immunity Cardiff University School of Medicine Cardiff UK; ^4^ Systems Immunity Research Institute Cardiff University School of Medicine Cardiff UK; ^5^ Institute of Immunology Faculty of Medicine Carl Gustav Carus Technische Universistät Dresden Dresden Germany; ^6^ National Center for Tumor Diseases University Hospital Carl Gustav Carus Technische Universistät Dresden Dresden Germany; ^7^ German Cancer Consortium (DKTK) Dresden Germany; ^8^ German Cancer Research Center (DKFZ) Heidelberg Germany; ^9^ Australian Institute of Health and Medical Research James Cook University Cairns QLD Australia

**Keywords:** cancer immunotherapy, CD141, CLEC9A, dendritic cells, vaccines, Wilms' tumor 1

## Abstract

**Objectives:**

Vaccines that prime Wilms' tumor 1 (WT1)‐specific CD8^+^ T cells are attractive cancer immunotherapies. However, immunogenicity and clinical response rates may be enhanced by delivering WT1 to CD141^+^ dendritic cells (DCs). The C‐type lectin‐like receptor CLEC9A is expressed exclusively by CD141^+^ DCs and regulates CD8^+^ T‐cell responses. We developed a new vaccine comprising a human anti‐CLEC9A antibody fused to WT1 and investigated its capacity to target human CD141^+^ DCs and activate naïve and memory WT1‐specific CD8^+^ T cells.

**Methods:**

WT1 was genetically fused to antibodies specific for human CLEC9A, DEC‐205 or β‐galactosidase (untargeted control). Activation of WT1‐specific CD8^+^ T‐cell lines following cross‐presentation by CD141^+^ DCs was quantified by IFNγ ELISPOT. Humanised mice reconstituted with human immune cell subsets, including a repertoire of naïve WT1‐specific CD8^+^ T cells, were used to investigate naïve WT1‐specific CD8^+^ T‐cell priming.

**Results:**

The CLEC9A‐WT1 vaccine promoted cross‐presentation of WT1 epitopes to CD8^+^ T cells and mediated priming of naïve CD8^+^ T cells more effectively than the DEC‐205‐WT1 and untargeted control‐WT1 vaccines.

**Conclusions:**

Delivery of WT1 to CD141^+^ DCs via CLEC9A stimulates CD8^+^ T cells more potently than either untargeted delivery or widespread delivery to all Ag‐presenting cells via DEC‐205, suggesting that cross‐presentation by CD141^+^ DCs is sufficient for effective CD8^+^ T‐cell priming in humans. The CLEC9A‐WT1 vaccine is a promising candidate immunotherapy for malignancies that express WT1.

## Graphical abstract

This project developed a new vaccine comprising a human anti‐CLEC9A antibody fused to the immunogenic, broadly expressed tumor antigen, WT1. This vaccine specifically delivered WT1 to human CD141^+^ (cDC1) dendritic cells, for efficient cross‐presentation and activation of naïve and memory WT1‐specific CD8^+^ T cells. The anti‐CLEC9A‐WT1 antibody is a promising new vaccine candidate for malignancies that express WT1.

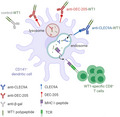

## Introduction

Relapse after initial treatment is a major problem in the clinical management of acute myeloid leukaemia (AML). Moreover, there are no effective treatments for myelodysplastic syndrome (MDS), which predisposes to AML. In these and other haematological malignancies, vaccines that prime and/or boost tumor‐specific CD8^+^ T cells are attractive therapeutic options to prevent or delay disease progression, especially in the setting of minimal residual disease (MRD).^1^, ^2^ As the initiators and orchestrators of adaptive immunity, dendritic cells (DCs) are essential for the induction of tumor‐specific CD8^+^ T‐cell responses.^3^, ^4^ DC‐based vaccines have been administered safely to thousands of cancer patients over the past two decades, with clear evidence of immunogenicity and some indications of clinical efficacy, most notably in patients with AML.^2^ Clinical response rates may nonetheless be improved by more potent vaccines.^1^, ^5^

The transcription factor Wilms' tumor 1 (WT1) is one of the best characterised and most highly immunogenic tumor‐associated antigens (TAAs) and is an excellent target for therapeutic cancer vaccines.^6^ WT1 is overexpressed in a range of haematological and solid malignancies, including most cases of AML.^7^ Indeed, peripheral blood measurements of WT1 mRNA are routinely used to detect and monitor MRD in patients with AML or MDS.^8^ WT1 is minimally expressed in somatic tissues, but it is expressed by leukaemic stem cells and plays an important role in leukaemogenesis, making it less prone to immune escape. WT1 is also highly immunogenic and contains epitopes restricted by multiple HLA allotypes that are recognised by CD4^+^ or CD8^+^ T cells.^7^, ^9^ Although peptide‐based vaccines can elicit functional WT1‐specific CD8^+^ T‐cell responses and show therapeutic efficacy in some patients,^10^, ^11^ they largely fail to elicit long‐term memory responses that incorporate high‐avidity WT1‐specific CD8^+^ T cells.^12^, ^13^ A more promising approach has been the development of cell‐based vaccines comprising autologous monocyte‐derived DCs (moDCs) loaded *in vitro* with WT1 mRNA, which have been shown to prevent and/or delay relapse after chemotherapy and improve overall survival in patients with high‐risk AML.^14^, ^15^ However, moDC‐based vaccines are expensive, labour‐intensive, and require specialist facilities, and greater immunogenicity may be achieved by targeting other subsets of DCs.^2^, ^3^, ^5^ An unmet clinical need therefore exists for improved ‘off‐the‐shelf’ vaccine formulations that elicit potent immune responses against WT1.

Antibodies (Abs) specific for antigen (Ag) uptake receptors are attractive candidates for the delivery of vaccine cargo directly to DCs *in vivo*.^3^, ^16^ For example, Abs specific for the C‐type lectin receptor DEC‐205 can deliver TAAs to DCs, which prime TAA‐specific CD4^+^ and CD8^+^ T‐cell responses and display greater therapeutic efficacy in mice than other vaccine formulations.^17^, ^18^, ^19^ Moreover, vaccination with a human DEC‐205‐specific Ab conjugated to NY‐ESO‐1 and combined with the adjuvant polyICLC was feasible and well tolerated in patients with advanced solid malignancies and MDS, and in some cases, enhanced NY‐ESO‐1‐specific CD4^+^ and CD8^+^ T‐cell responses were observed after vaccination.^20^, ^21^ In addition, DEC‐205‐specific Ab‐based vaccines can be combined with standard treatments, including doxorubicin and/or decitabine,^21^, ^22^ and may benefit patients receiving checkpoint inhibitors.^20^

Dendritic cells comprise several distinct subsets with highly specialised roles in the generation of specific immune responses.^4^ In humans, DEC‐205 is widely expressed by most leucocytes, including all DCs.^23^ Vaccine immunogenicity may therefore be enhanced by specifically targeting particular subtypes of DCs. In mice, CD8^+^ T‐cell‐mediated control of various tumors relies on the activity of conventional type 1 dendritic cells (cDC1s), which have also been identified as Batf3‐dependent, CD103^+^ tissue‐resident, and CD8α^+^ lymphoid‐resident DCs.^24^, ^25^, ^26^, ^27^ Moreover, cDC1s underpin the efficacy of various cancer therapies, including adoptive T‐cell transfer,^28^, ^29^ radiotherapy, immunogenic and oncolytic virus‐based interventions,^30^, ^31^ agonistic anti‐CD137 Abs,^32^ and immune checkpoint inhibitors.^32^, ^33^ The human cDC1 equivalents are known as CD141^+^ DCs.^34^, ^35^, ^36^, ^37^ They are more effective than moDCs at cross‐presenting cellular Ags, a key process in the generation of tumor‐specific CD8^+^ T‐cell responses.^34^, ^38^ Moreover, the presence of CD141^+^ DCs in many human solid tumors correlates positively with prolonged survival and enhanced responses to anti‐PD‐1 therapy.^28^, ^29^, ^39^, ^40^

The C‐type lectin‐like receptor CLEC9A, also called DNGR‐1, is an attractive target for vaccine enhancement, because it is exclusively expressed by CD141^+^ DCs and plays a critical role in the recognition and cross‐presentation of necrotic cells Ags.^41^, ^42^, ^43^, ^44^, ^45^ Anti‐mouse CLEC9A Abs are as effective as anti‐mouse DEC‐205 Abs at delivering Ags to murine cDC1s *in vivo* to prime CD8^+^ T‐cell responses,^46^ as well as humoral and CD4^+^ T‐cell responses, which collectively mediate protective tumor‐specific immunity.^41^, ^42^ We previously developed vaccines comprising anti‐human CLEC9A or anti‐human DEC‐205 IgG4 Abs genetically fused to a long peptide (40 amino acids) from the human cytomegalovirus (CMV) pp65 Ag.^47^ Despite similar uptake and internalisation of these anti‐CLEC9A and anti‐DEC‐205 Abs by CD141^+^ DCs, and a comparable ability to stimulate CMV‐specific memory CD4^+^ T‐cell responses, the anti‐CLEC9A Ab more effectively targeted the cross‐presentation pathway in CD141^+^ DCs, leading to greater activation of pp65‐specific memory CD8^+^ T cells in HLA class I transgenic NOD/SCID/IL2rgKO (NSG) mice. However, it is unclear if similarly beneficial effects could be elicited in humans by exclusively targeting TAA to the rare CD141^+^ DC subset via CLEC9A.

In this study, we developed chimeric vaccines comprising anti‐human CLEC9A or anti‐human DEC‐205 IgG4 Abs genetically fused to a polypeptide from WT1. The CLEC9A‐WT1 vaccine more effectively promoted cross‐presentation of HLA‐A*0201‐restricted and HLA‐A*2402‐restricted WT1 epitopes by CD141^+^ DCs, leading to greater activation of WT1‐specific CD8^+^ T cells. Using a novel humanised mouse model in which human DC subsets develop *in vivo*, we also found that the CLEC9A‐WT1 vaccine effectively primed naïve CD8^+^ T cells. Collectively, these data identify a promising new vaccine candidate for malignancies that express WT1.

## Results

### Generation and validation of human CLEC9A‐WT1 and DEC‐205‐WT1 vaccines

**Figure 1:**
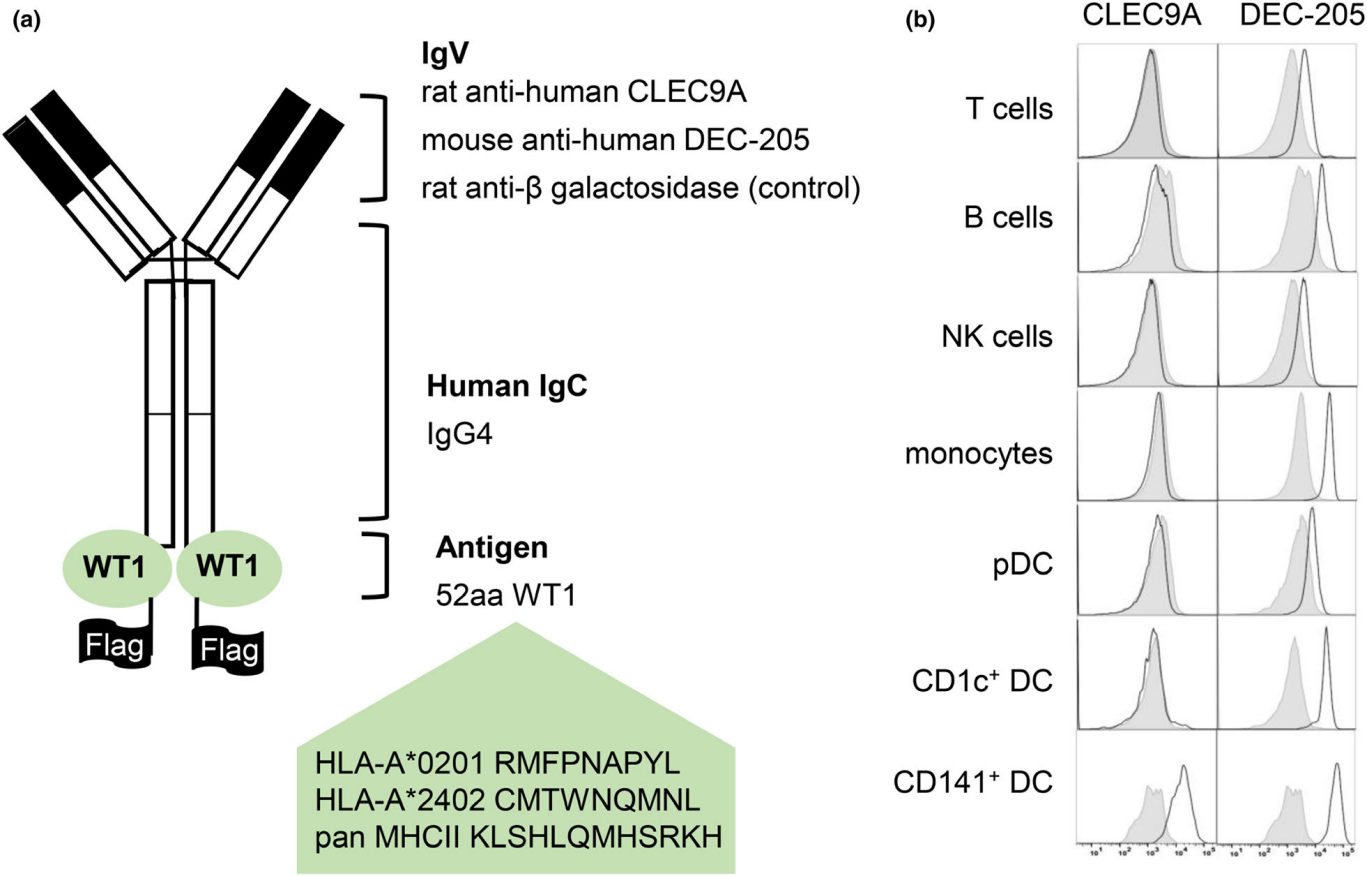
Generation and validation of human CLEC9A‐WT1 and DEC‐205‐WT1 vaccines. **(a)** Diagram of a chimeric Ab comprising rat or mouse variable regions specific for human CLEC9A, human DEC‐205, or bacterial β‐galactosidase (β‐gal), and human IgG4 and *κ* constant regions genetically fused to an antigenic sequence from WT1 containing the HLA‐A*201‐restricted WT1_126–134_ (RMFPNAPYL) and HLA‐A*2402‐restricted WT1_235–243_ (CMTWNQMNL) epitopes, a pan‐MHC II epitope (KLSHLQMHSRKH), and a FLAG tag. **(b)** Flow cytometric analysis of CLEC9A‐WT1 (white, left panels), DEC‐205‐WT1 (white, right panels) and control‐WT1 (grey, control) binding to human PBMCs. Data are representative of three healthy blood donors.

We previously developed constructs encoding human chimeric IgG4 Abs specific for CLEC9A, DEC‐205, and β‐gal (untargeted isotype control).^47^ The WT1 sequence, encoding both the HLA‐A*2402‐restricted WT1_235–243_ epitope and the HLA‐A*0201‐restricted WT1_126–134_ epitope, was genetically fused to the heavy chain of each Ab via an alanine linker, so that each Ab contained two WT1 antigenic sequences (Figure 1a). After expression in 293F cells and purification by affinity chromatography, Ab binding specificity to target receptors was confirmed on CLEC9A‐transfected or DEC‐205‐transfected cell lines by ELISA and further evaluated on PBMCs by flow cytometry (Figure 1b and Supplementary figure 1). As expected, CLEC9A‐WT1 bound specifically to CD141^+^ DCs, whereas DEC‐205‐WT1 bound to most human leucocyte subsets, with the highest staining intensity observed on CD1c^+^ DCs and CD141^+^ DCs (Figure 1b).

### Cross‐presentation of WT1 epitopes by CD141^+^ DCs after uptake of CLEC9A‐WT1

**Figure 2:**
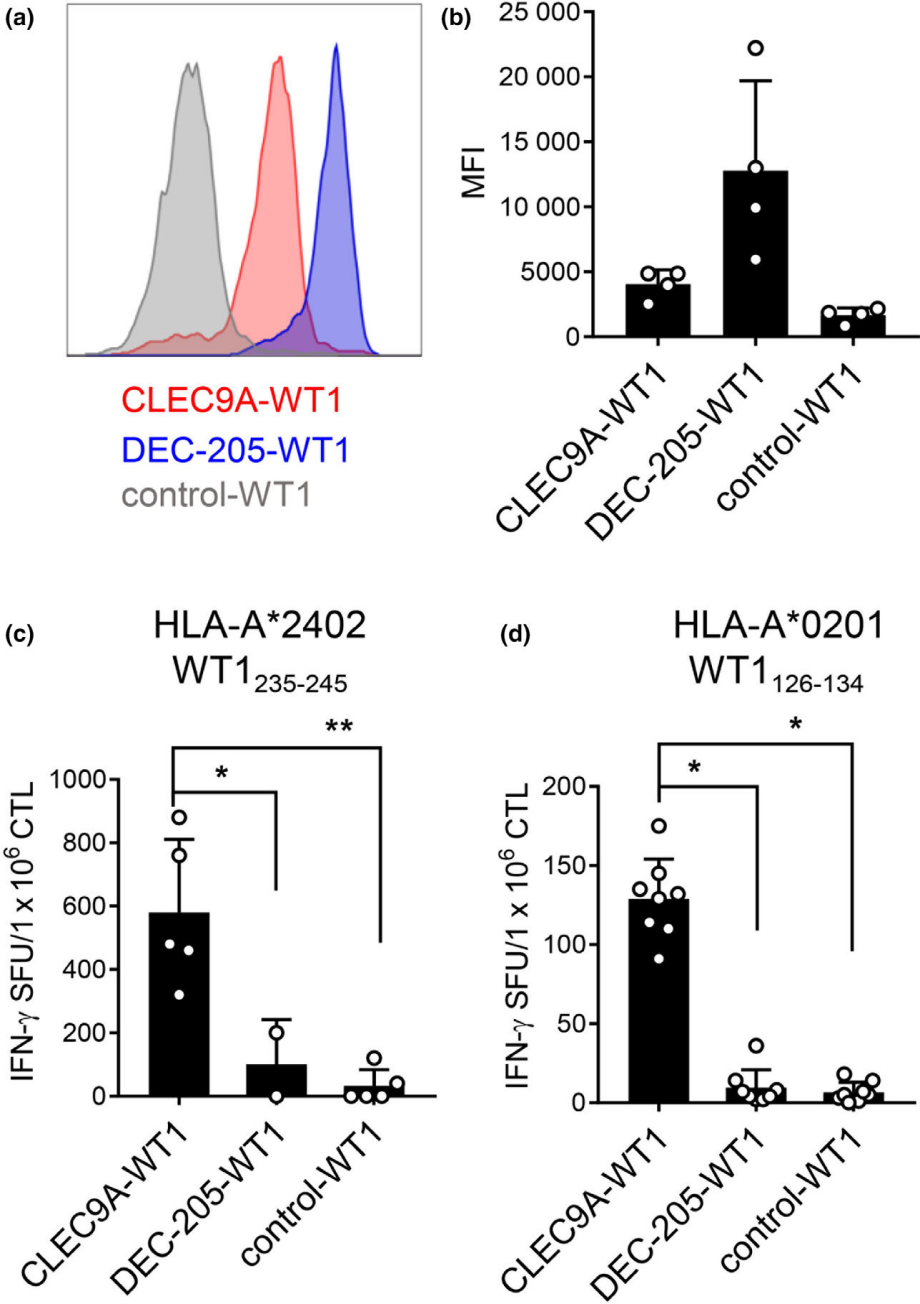
Cross‐presentation of WT1 epitopes by CD141^+^ DCs after uptake of CLEC9A‐WT1. **(a, b)** Binding of CLEC9A‐WT1, DEC‐205‐WT1 and β‐gal‐WT1 (control) Abs to *in vitro* differentiated CD141^+^ DCs. **(a)** Histograms from one representative donor. **(b)** Median fluorescence intensity (MFI) mean + SD from four donors. **(c)** Cross‐presentation of the WT1_235–243_ epitope to WT1_235–243_‐specific CD8^+^ T cells by HLA‐A*2402^+^ CD141^+^ DCs cultured with CLEC9A‐WT1, DEC‐205‐WT1 or β‐gal‐WT1 (control). Data are shown as mean + SD (five donors). CTL, cytotoxic T lymphocyte; SFU, spot‐forming unit (IFNγ ELISPOT assay). *P = 0.0173, **P = 0.0014 (one‐way ANOVA with Tukey's test for multiple comparisons). **(d)** Cross‐presentation of the WT1_126–134_ epitope to WT1_126–134_‐specific CD8^+^ T cells by HLA‐A*0201^+^ CD141^+^ DCs cultured with CLEC9A‐WT1, DEC‐205‐WT1 or β‐gal‐WT1 (control). Data shown are mean + SD (pooled replicates from three donors). CTL, cytotoxic T lymphocyte; SFU, spot‐forming unit (IFNγ ELISPOT assay). *P < 0.0001 (one‐way ANOVA with Tukey's test for multiple comparisons).

As CD141^+^ DCs are extremely rare in human blood, we derived these cells from human cord blood CD34^+^ HSCs, either *in vitro* (Supplementary figure 2) using a previously validated culture system^48^ or *in vivo* using a humanised mouse model.^49^, ^50^, ^51^ The functional, phenotypic, and transcriptomic properties of the CD141^+^ DCs that emerge in each system closely resemble those of their naturally occurring counterparts.^48^, ^49^, ^50^, ^51^ The CLEC9A‐WT1 and DEC‐205‐WT1, but not the control‐WT1 vaccine, bound to *in vitro*‐derived CD141^+^ DCs. Of note, DEC‐205‐WT1 stained with greater intensity than CLEC9A‐WT1 in all donors, although this trend did not reach statistical significance (Figure 2a and b). The CLEC9A‐WT1 vaccine also facilitated cross‐presentation of the HLA‐A*2402‐restricted WT1_235–243_ epitope by purified CD141^+^ DCs to WT1_235–243_‐specific CD8^+^ T cells (Figure 2c, Supplementary figure 3). Similarly, efficient cross‐presentation of the HLA‐A*0201‐restricted WT1_126–134_ epitope to specific CD8^+^ T cells was observed after incubation of HLA‐A*0201^+^ CD141^+^ DCs with the CLEC9A‐WT1 vaccine (Figure 2d). In contrast, the DEC‐205‐WT1 and control‐WT1 vaccines were ineffective at delivering WT1 to CD141^+^ DCs for cross‐presentation of either the WT1_126–134_ epitope or the WT1_235–243_ epitope (Figure 2c and d).

### DCs and naïve WT1‐specific CD8^+^ T cells develop in humanised mice

**Figure 3:**
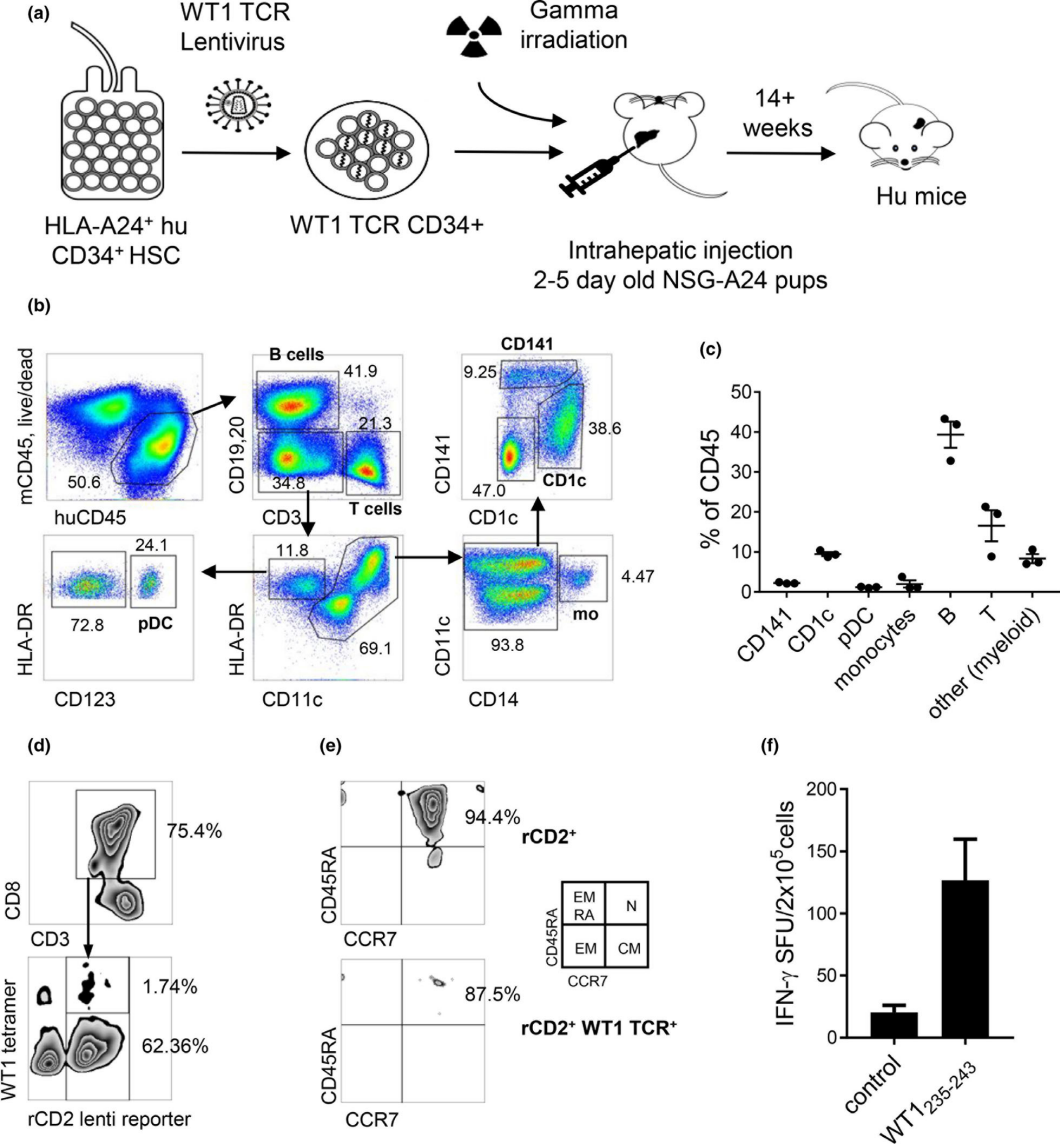
DCs and naïve WT1‐specific CD8^+^ T cells develop in humanised mice. **(a)** Diagram showing the generation of humanised NSG‐A24 mice using cord blood‐derived HSCs transduced with a lentivirus expressing a WT1_235–243_‐specific TCR. **(b, c)** Human CD45^+^ cell reconstitution in spleens from humanised NSG‐A24 mice. **(b)** Representative dot plots. **(c)** Human CD45^+^ cell subset composition (Means ± SD from three mice). **(d)** Expression of the WT1_235–243_ **‐**specific TCR on splenic human naïve CD3^+^ CD8^+^ T cells, detected using the corresponding tetramer and rat CD2. **(e)** Phenotype of CD3^+^ CD8^+^ T cells, gated on total transduced cells (rCD2^+^, top) or transduced cells expressing the WT1_235–243_‐specific TCR (bottom). CM, central memory (CD45RA^−^ CCR7^+^); EM, effector memory (CD45RA^−^ CCR7^−^); EMRA, terminally differentiated effector memory (CD45RA^+^ CCR7^−^); N, naïve (CD45RA^+^ CCR7^+^). **(f)** IFNγ production after incubation of humanised mouse splenocytes with WT1_235–243_ peptide (ELISPOT assay). Data represent pooled replicates from five humanised mice.

To investigate the *in vivo* effects of CLEC9A‐WT1 and DEC‐205‐WT1 vaccines, we generated humanised mice reconstituted with human immune cell subsets, including a small repertoire of naïve WT1_235–243_‐specific CD8^+^ T cells (Figure 3). Initially, HLA‐A*2402^+^ human HSCs were transduced with a lentivirus expressing a prearranged WT1_235–243_‐specific TCR and the reporter gene rat CD2. These transduced HSCs were then administered to immunodeficient NSG‐A24 neonatal mice (Figure 3a). After 10–14 weeks, human T cells, B cells, monocytes, and DC subsets were reconstituted in the spleens of humanised mice at frequencies similar to those reported previously^49^, ^52^ (Figure 3b and c). The functional, phenotypic, and transcriptomic properties of human DC subsets generated in humanised mice closely resemble those of their human peripheral blood counterparts.^49^, ^50^, ^51^ Moreover, transduction of the TCR transgene into human CD34^+^ HSCs before the development of CD8^+^ T cells *in vivo* enables thymic selection of human HLA‐A*2402^+^ CD8^+^ T cells in NSG‐A24 mice. Introduction of the TCR transgene also inhibits endogenous TCR gene rearrangement, resulting in exclusive expression of the transgenic TCR on the cell surface via a process known as allelic exclusion.^53^ An average of 13.17% ± 26.34% of engrafted human CD8^+^ T cells in the peripheral blood expressed rat CD2, indicative of successful integration of the transgene, but only a small proportion (< 2%) expressed detectable cell surface levels of the WT1_235–243_‐specific TCR (Figure 3d). All of the transduced CD8^+^ T cells in these humanised mice coexpressed CD45RA and CCR7, consistent with a naïve phenotype (Figure 3e). The expressed WT1_235–243_‐specific TCR also transduced a functional signal, leading to the production of IFNγ after stimulation of humanised mouse splenocytes with the cognate WT1_235–243_ peptide (Figure 3f).

### CLEC9A‐WT1 induces priming of human naïve WT1‐specific CD8^+^ T cells

**Figure 4:**
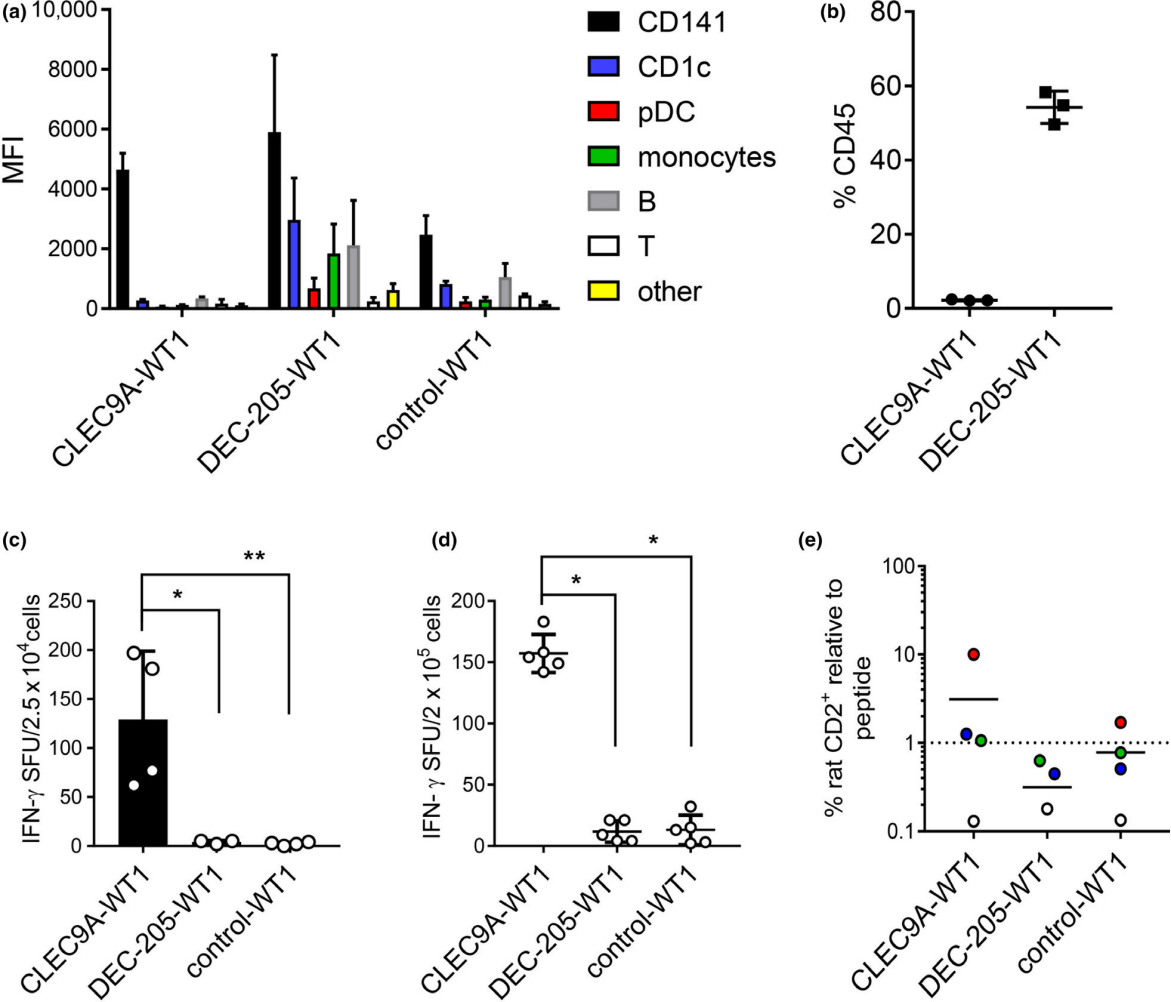
CLEC9A‐WT1 induces priming of human naïve WT1‐specific CD8^+^ T cells. **(a, b)** Binding of CLEC9A‐WT1, DEC‐205‐WT1 and β‐gal‐WT1 (control) to humanised mouse splenocytes. **(a)** Median fluorescence intensity (MFI) across human immune cell subsets. **(b)** Per cent frequency among total human CD45^+^ cells. Data are shown as mean + SD (three humanised mice). **(c)** Cross‐presentation of the WT1_235–243_ epitope to WT1_235–243_ **‐**specific CD8^+^ T cells by HLA‐A*2402^+^ CD141^+^ DCs flow‐purified from bone marrow of humanised mice after culture with CLEC9A‐WT1, DEC‐205‐WT1 or β‐gal‐WT1 (control). SFU, spot‐forming unit (IFNγ ELISPOT assay). Data are shown as mean + SD (four humanised mice). *P = 0.0121, **P = 0.0073 (one‐way ANOVA with Tukey's test for multiple comparisons). **(d)** Priming of naïve WT1_235–243_‐specific CD8^+^ T cells after culture of unfractionated human CD45^+^ cells from humanised mouse spleens with CLEC9A‐WT1, DEC‐205‐WT1 or β‐gal‐WT1 (control). Data are shown as mean + SD (five humanised mice). SFU, spot‐forming unit (IFNγ ELISPOT assay). *P < 0.0001 (one‐way ANOVA with Tukey's test for multiple comparisons). **(e)** Expansion of rat CD2^+^ WT1_235–243_‐specific TCR^+^ CD8^+^ T cells in humanised mouse splenocytes 8 days after an initial 2 h prime with CLEC9A‐WT1, DEC‐205‐WT1 or β‐gal‐WT1 (control) in the presence of activators poly I:C + R848. Data are expressed as fold expansion normalised to expansion after culture with cognate peptide (dotted line). Each colour represents cultures from the same humanised mouse. Horizontal bars show mean values (four humanised mice).

The capacity of DEC‐205‐WT1 to bind other DC and leucocyte subsets (Figure 1b) raises the possibility that inefficient cross‐presentation by monocytes, plasmacytoid DCs (pDCs) and CD1c^+^ DCs as well as CD141^+^ DCs could be as effective at priming CD8^+^ T cells *en masse* as highly efficient cross‐presentation by only CD141^+^ DCs targeted with CLEC9A‐WT1. To address this question, we first confirmed binding of CLEC9A‐WT1, DEC‐205‐WT1 and control‐WT1 vaccines to human immune cell populations that developed in the spleens of humanised mice (as defined in Figure 3b and c). As expected, CLEC9A‐WT1 bound specifically to CD141^+^ DCs (Figure 4a), which constituted only 2% of the total engrafted human CD45^+^ population (Figure 4b). DEC‐205‐WT1 also bound to CD141^+^ DCs and, to a lesser extent, to CD1c^+^ DCs, B cells and monocytes, which together represented more than 50% of the human CD45^+^ cell population (Figure 4a and b). Despite largely equivalent binding of CLEC9A‐WT1 and DEC‐205‐WT1 to humanised mouse CD141^+^ DCs and a small amount of non‐specific binding of control‐WT1 to humanised mouse CD141^+^ DCs (Figure 4a), cross‐presentation of the WT1_235–243_ epitope by purified humanised mouse CD141^+^ DCs to WT1_235–243_‐specific CD8^+^ T cells was only detectable after incubation with CLEC9A‐WT1 (Figure 4c). This finding confirmed and extended our observations with *in vitro* cultured CD141^+^ DCs (Figure 2c).

To determine whether human naïve CD8^+^ T cells could be primed by specific targeting of CD141^+^ DCs via CLEC9A and/or broad targeting of more abundant Ag‐presenting cells via DEC‐205, we incubated bulk human CD45^+^ leucocytes from the spleens of humanised mice containing naïve WT1_235–243_‐specific CD8^+^ T cells (Figure 3a–f) with CLEC9A‐WT1, DEC‐205‐WT1 and control‐WT1 vaccines and monitored the activation of CD8^+^ T cells using an IFNγ ELISPOT assay (Figure 4d). In this model, only the CLEC9A‐WT1 vaccine effectively stimulated IFNγ production (Figure 4d). We also examined whether CLEC9A‐WT1 could induce the expansion of naïve WT1_235–243_‐specific CD8^+^ T cells (Figure 4e). In three of four donors, CLEC9A‐WT1 induced the expansion of rat CD2^+^ WT1_235–243_‐specific TCR^+^ CD8^+^ T cells over a period of 8 days in culture to a similar or greater extent than stimulation with the cognate peptide (Figure 4e and Supplementary figure 4). In contrast, DEC‐205‐WT1 and control‐WT1 vaccines failed to induce the expansion of WT1_235–243_‐specific CD8^+^ T cells, with the exception of one donor population incubated with the control‐WT1 vaccine (Figure 4e). Collectively, these data show that specific delivery of Ag to CD141^+^ DCs via CLEC9A stimulates CD8^+^ T cells more potently than either untargeted delivery or widespread delivery to all Ag‐presenting cells via DEC‐205.

## Discussion

In this study, we report the development of a human CLEC9A‐WT1 vaccine that specifically delivers WT1 to CD141^+^ DCs, which efficiently cross‐present HLA‐A*0201‐restricted and HLA‐A*2402‐restricted epitopes derived from WT1. We found that targeting CD141^+^ DCs with CLEC9A‐WT1 resulted in more effective cross‐presentation of these WT1 epitopes compared with either an untargeted control‐WT1 vaccine or an equivalent DEC‐205‐WT1 vaccine, which bound efficiently to CD141^+^ DCs and more abundant subtypes of DCs. Moreover, we showed that the CLEC9A‐WT1 vaccine primed naïve WT1‐specific CD8^+^ T cells more effectively than the control‐WT1 or DEC‐205‐WT1 vaccines. These data identify CLEC9A‐WT1 as a promising new vaccine candidate for malignancies that express WT1.

Our CLEC9A‐WT1 vaccine offers several key advantages over existing cancer vaccines that have already shown promise in early clinical trials. First, it can be produced as an ‘off‐the‐shelf’ clinical‐grade formulation, which circumvents the financial and logistical issues associated with *in vitro*‐manufactured patient‐specific vaccines. Second, this prototype vaccine targets very precisely the key DC subset required for the initiation of tumor‐specific immune responses, thereby maximising potential efficacy while minimising off‐target effects.^3^, ^16^ Moreover, the human IgG4 *κ* backbone contains two point mutations (S229P and L236E) that enhance stability and minimise elimination via Ab‐dependent cellular cytotoxicity (ADCC) or complement‐dependent cytotoxicity (CDC), making it suitable as a therapeutic agent.^47^ Of note, the two United States Food and Drug Administration (FDA)‐approved anti‐PD‐1 checkpoint inhibitor Abs, pembrolizumab and nivolumab, are similarly structured IgG4 Abs. The human IgG4 isotype, combined with the specificity of CLEC9A‐WT1 for CD141^+^ DCs, therefore favors retention in the bloodstream, enhancing the longevity of exposure and the potential for T‐cell activation. Third, CLEC9A trafficks Ags to early endosomal compartments, where cross‐presentation is most efficient.^54^ This critical function likely explains the greater efficacy of CLEC9A‐WT1 compared with DEC‐205‐WT1. Fourth, our chimeric Ab vaccine delivers WT1, which is currently recognised as one of the most immunogenic TAAs. WT1 is widely expressed by many human cancers and contains numerous CD4^+^ and CD8^+^ T‐cell epitopes distributed across multiple HLA allotypes.^6^ It is also expressed by cancer stem cells and plays a key role in oncogenesis, making it an ideal immunotherapeutic target in many cancer patients.

Efficient cross‐presentation by CD141^+^ DCs after uptake of CLEC9A‐WT1 *in vitro* did not require prior activation, consistent with our previous work.^47^ However, adjuvants are known to enhance T‐cell priming induced by vaccines that target DCs, at least in mouse models, and the same may be true for CLEC9A‐WT1. In this regard, polyICLC is safe to administer in combination with DEC‐205‐NY‐ESO‐1 and can effectively activate CD141^+^ DCs, making this adjuvant a logical choice to combine with CLEC9A‐WT1.^20^, ^21^, ^34^ The route of vaccine administration is yet to be addressed but may not be optimal via the dermal route given the reported lack of surface expression of CLEC9A by dermal DC.^55^ Another consideration is that systemic impairments in haematopoiesis in advanced cancer patients can impact DC numbers, although there is scant literature on the functional and numerical integrity of CD141^+^ DCs under these circumstances. MoDC‐based vaccines have shown the greatest clinical benefit in high‐risk AML patients with MRD.^14^ Although CD141^+^ DCs are depleted in patients with newly diagnosed AML and in patients with relapse/refractory AML, they recover in the setting of MRD after chemotherapy or allogeneic stem cell transplantation,^56^ suggesting that vaccination with CLEC9A‐WT1 will not only be feasible, but will also likely be most efficacious in this setting as an adjunctive treatment for AML. WT1 peptide, protein and moDC‐based vaccines have also been administered safely to patients with MDS, chronic myeloid leukaemia, non‐Hodgkin's lymphoma, multiple myeloma, paediatric leukaemias and advanced solid malignancies including breast, lung, renal, ovarian, and pancreatic cancers, and glioblastoma.^7^, ^10^, ^57^, ^58^, ^59^, ^60^, ^61^, ^62^, ^63^ CLEC9A‐WT1 therapy may therefore be suitable for patients with any of these malignancies, and any defects in CD141^+^ DC numbers could be overcome by the administration of Flt3L.^64^ Mouse cDC1s play a key role in responses to radiation therapy, viral therapies, adoptive T‐cell therapies and immune checkpoint inhibitors.^25^, ^26^, ^28^, ^29^, ^30^, ^31^, ^40^, ^65^, ^66^ Accordingly, CLEC9A‐WT1 vaccines also hold the potential to enhance immunogenicity and response rates when combined with a variety of immunotherapies and standard treatments in patients with cancers that express WT1.

The generation of comparable CLEC9A‐WT1 and DEC‐205‐WT1 vaccines on the same IgG4 backbone in this study enabled a direct comparison of two distinct specificities targeting the same Ag to CD141^+^ DCs. Superior cross‐presentation of the HLA‐A*0201‐restricted and HLA‐A*2402‐restricted WT1 epitopes was observed with CLEC9A‐WT1, despite equivalent or superior binding of DEC‐205‐WT1 to CD141^+^ DCs. A similar result was obtained previously with an immunodominant HLA‐A*0201‐restricted pp65 epitope from CMV.^47^ This phenomenon may be explained by the fact that DEC‐205 trafficks to lysosomes, whereas CLEC9A localises to early endosomes, which favor cross‐presentation.^16^, ^54^, ^67^ These data emphasise the choice of receptor as an important consideration in vaccine design and show that Ag delivery to the cross‐presentation pathway can enhance the immunogenicity of specific formulations that target CD141^+^ DCs.

To compare the priming capabilities of CLEC9A‐WT1 and DEC‐205‐WT1, we generated humanised mice reconstituted with human immune cell subsets, including a small repertoire of naïve WT1_235–243_‐specific CD8^+^ T cells. Using splenocytes from these mice*,* we demonstrated that CLEC9A‐WT1 can induce CD141^+^ DC‐mediated priming of human naïve WT1‐specific T cells, in contrast to DEC‐205‐WT1. These data apparently conflict with a previous study in which both anti‐CLEC9A and anti‐DEC‐205 Abs were able to prime murine CD8^+^ T cells by preferentially targeting cDC1s.^46^ Our model nonetheless supports the notion that effective CD8^+^ T‐cell priming in humans can be achieved by specific targeting of Ag to CD141^+^ DCs.

It is important to note that Ag‐presenting cells other than CD141^+^ DCs can prime CD8^+^ T cells, and the extent to which this occurs in humans may not have been fully revealed using the DEC‐205‐WT1 vaccine. For example, human CD1c^+^ DCs, which are more abundant than CD141^+^ DCs and equivalent to mouse cDC2s, do not efficiently cross‐present Ags after delivery via DEC‐205.^16^, ^67^ In contrast, equivalent levels of cross‐presentation were observed *in vitro* with human CD1c^+^ DCs and human CD141^+^ DCs after Ag delivery via CD40, which trafficks to early endosomes, like CLEC9A.^67^, ^68^ Anti‐DEC‐205 and anti‐CD40 Abs conjugated to influenza matrix protein M1 epitopes nonetheless reactivated M1‐specific memory CD8^+^ T cells to a similar extent in a humanised mouse model, suggesting that cross‐presentation by both CD1c^+^ DCs and CD141^+^ DCs is unlikely to enhance CD8^+^ T‐cell responses *in vivo*.^68^ In addition, both CD1c^+^ DCs and CD141^+^ DCs process and present epitopes to reactivate memory CD4^+^ T cells after Ag delivery via DEC‐205.^47^ Consequently, Ag delivery to CD1c^+^ DCs may further enhance tumor‐specific CD4^+^ T‐cell responses, even though anti‐CLEC9A and anti‐DEC‐205 Abs promote CD4^+^ T‐cell responses similarly after uptake by CD141^+^ DCs.^69^ Chimeric anti‐CLEC9A and anti‐DEC‐205 Abs also prime HIV‐specific CD4^+^ T‐cell responses to a similar extent in mice.^46^ In mice and macaques, CLEC9A Ab targeting also induces potent humoral responses, even in the absence of adjuvant.^70^ Although this is yet to be explored in humans, the potential for high titre Ab responses could be a limitation to multiple treatment cycles that may be required for enhanced cellular immunity. Targeting a single known TAA is a further potential limitation of this vaccine; however, conjugation of other TAA individually or in combination should also be feasible. Further work is therefore required to assess the overall immunogenicity and clinical efficacy of different chimeric Abs, which collectively provide a generic platform to develop novel vaccines against numerous cancers and infectious agents.

Our data show that a CLEC9A‐WT1 vaccine selectively targets CD141^+^ DCs, leading to enhanced cross‐presentation and activation of naïve and memory WT1‐specific CD8^+^ T cells. Collectively, these findings identify a promising new vaccine candidate for malignancies that express WT1.

## Methods

### Blood samples

Peripheral venous blood was obtained from healthy adult volunteers, and umbilical cord blood (UCB) was obtained from the Queensland Cord Blood Bank. Written informed consent was obtained for sample acquisition in line with standards established by the Declaration of Helsinki. Study approval was granted by the Mater Human Research Ethics Committee (HREC13/MHS/83 and HREC13/MHS/86). CD34^+^ haematopoietic stem cells (HSCs) were isolated from UCB using a CD34^+^ Isolation Kit (Miltenyi Biotec, Bergisch Gladbach, North Rhine‐Westphalia, Germany) and cryopreserved at −80 °C. Typing for HLA‐A*0201 and HLA‐A*2402 was confirmed by PCR.^52^, ^71^

### Generation and validation of human CLEC9A‐WT1 and DEC‐205‐WT1 vaccines

Expression constructs were generated in pcDNA3.1^+^ plasmids encoding the human IgG4 and *κ* constant regions and the variable regions of rat anti‐human CLEC9A clone 4C6, mouse anti‐human DEC‐205 clone MMRI‐7, or rat anti‐bacterial β‐galactosidase (β‐gal) clone GL117.^47^ The antibody heavy chain sequence was fused to cDNA encoding an alanine linker, the WT1 antigenic sequence AAAASSGQARMFPNAPYLPSSQLECMTWNQMNLGACNKRYFKLSHLQMHSRKHTGSGSGDYKDDDDK (corresponding to amino acid positions 124‐136, 231‐245, 330‐349 of WT1, isoform CRA_a; Sequence ID: EAW68220.1), a serine‐glycine linker, and a FLAG tag to facilitate purification and detection of the chimeric Ab (GeneArt Thermo Fisher Scientific, Waltham, MA, USA). The resulting chimeric Abs, each carrying two WT1 antigenic sequences, were expressed in mycoplasma‐free Freestyle 293F cells (Invitrogen Thermo Fisher Scientific, Waltham, MA, USA) using 293fectin (Invitrogen Thermo Fisher Scientific, Waltham, MA, USA) and purified from the culture supernatant by affinity chromatography using Protein A Sepharose (GE Healthcare, Chicago, IL, USA). Ab specificity was confirmed by binding to 293F cells transiently transfected with full‐length recombinant CLEC9A or DEC‐205 and by binding to recombinant CLEC9A, DEC‐205 or β‐gal recombinant proteins by ELISA.^47^

To assess binding specificity to human peripheral blood mononuclear cell (PBMC) subsets, PBMCs were isolated from adult blood by density gradient centrifugation using Ficoll‐Paque (GE Healthcare), and a fraction of these cells were enriched using an Easysep Human Myeloid DC Enrichment Kit (Stemcell Technologies, Vancouver, BC, Canada). PBMCs and enriched myeloid DCs were blocked with 10% mouse serum and 10% rat serum (Sigma‐Aldrich, Saint Louis, MO, USA) and incubated with CLEC9A‐WT1, DEC‐205‐WT1 or β‐gal‐WT1 (control‐WT1) vaccines (Monash University, Clayton, VIC, Australia), followed by anti‐human IgG4‐biotin (Invitrogen Thermo Fisher Scientific). PBMCs were then stained with streptavidin‐PE (SA‐PE, Invitrogen Thermo Fisher Scientific), Live/Dead (Zombie) Aqua (BioLegend, San Diego, CA, USA), and anti‐CD3‐BV711 (clone OKT3), anti‐CD19‐Pacific Blue (clone HIB19), anti‐CD20‐Pacific Blue (clone 2H7), anti‐CD56‐FITC (clone HCD56), anti‐CD14‐APC (clone HCD14), anti‐CD16‐BV785 (clone 3G8), anti‐HLA‐DR‐PerCP‐Cy5.5 (clone L243), anti‐CD11c‐PE‐CF594 (clone B‐LY6, BD Biosciences, San Jose, CA, USA) and anti‐CD123‐BUV395 (clone 7G3, BD Biosciences). Enriched myeloid DCs were stained with SA‐PE (Invitrogen Thermo Fisher Scientific), Live/Dead Aqua (BioLegend), anti‐CD141‐PE‐Cy7 (clone M80), anti‐CD1c‐Alexa Fluor 700 (clone L161), anti‐HLA‐DR‐PerCP‐Cy5.5 (clone L243), and the lineage‐specific Abs anti‐CD3‐Pacific Blue (clone OKT3), anti‐CD14‐Pacific Blue (clone HCD14), anti‐CD16‐Pacific Blue (clone 3G8), anti‐CD19‐Pacific Blue (clone HIB19), anti‐CD20‐Pacific Blue (clone 2H7) and anti‐CD56‐Pacific Blue (clone HCD56). All Abs were purchased from BioLegend unless stated otherwise. Data were acquired using an LSRFortessa X‐20 (BD Biosciences) or a CytoFLEX‐S flow cytometer (Beckman Coulter, Indianapolis, IN, USA) and analysed using FlowJo software version 9 or 10 (Tree Star, Inc., Ashland, OR, USA).

### Expansion and differentiation of human CD141^+^ DCs *in vitro*

CD34^+^ HSCs were expanded and differentiated into DCs using a previously reported method with modifications.^48^ Briefly, CD34^+^ HSCs were expanded for 7 days in StemSpan medium (Stemcell Technologies) supplemented with 10% fetal bovine serum (FBS, Gibco Thermo Fisher Scientific), FMS‐like tyrosine kinase 3 ligand (Flt3L, 100 ng mL^−1^), stem cell factor (SCF, 100 ng mL^−1^), interleukin‐3 (IL‐3, 20 ng mL^−1^) and thrombopoietin (TPO, 50 ng mL^−1^) (all from PeproTech, Rocky Hill, NJ, USA), together with StemRegenin‐1 (SR‐1, 1 µm, Stemcell Technologies). HSCs were then differentiated into DCs for 10–11 days in RPMI 1640 medium (Gibco Thermo Fisher Scientific) supplemented with 10% FBS (Gibco Thermo Fisher Scientific), HEPES (10 mm), sodium pyruvate (1 mm), penicillin/streptomycin (100 U mL^−1^) and GlutaMAX (2 mm) (all from Gibco Thermo Fisher Scientific), together with β‐mercaptoethanol (50 µm, Sigma‐Aldrich), FLt3L (100 ng mL^−1^, PeproTech), SCF (20 ng mL^−1^, PeproTech), IL‐4 (2.5 ng mL^−1^, Invitrogen Thermo Fisher Scientific), GM‐CSF (2.5 ng mL^−1^, Invitrogen Thermo Fisher Scientific) and StemRegenin‐1 (SR‐1; 1 µm, Stemcell Technologies). DCs were phenotyped by staining the cultures with Live/Dead Aqua (BioLegend), lineage‐specific Abs conjugated to Pacific Blue (see above), and anti‐HLA‐DR‐PE‐Cy7 (clone L243), anti‐CD11c‐PE‐CF594 (clone B‐ly6, BD Biosciences), anti‐CD141‐BV711 (clone 1A4, BD Biosciences), anti‐CADM1‐Alexa Fluor 647 (clone 3E1, MBL, Woburn, MA, USA) and anti‐CLEC9A‐PE (clone 8F9). For binding assays, cells were incubated with CLEC9A‐WT1, DEC‐205‐WT1 or control‐WT1 Abs, followed by anti‐human IgG4‐biotin (Invitrogen Thermo Fisher Scientific), and then the DC phenotyping Ab cocktail (see above) with SA‐PE (Invitrogen Thermo Fisher Scientific). Data were acquired using an LSRFortessa X‐20 (BD Biosciences) and analysed using FlowJo software version 9 or 10 (Tree Star Inc.). In some experiments, CD141^+^ DCs were flow‐purified from the cultures using a MoFlo Astrios (Beckman Coulter) after staining with Live/Dead Aqua (BioLegend), lineage‐specific Abs conjugated to Pacific Blue (see above), and anti‐HLA‐DR‐PE‐Cy7 (clone L243), anti‐CD11c‐PE‐CF594 (clone B‐ly6, BD Biosciences) and anti‐CD141‐APC (clone 1A4, BD Biosciences).

### Humanised mice

All animal experiments were approved by the University of Queensland Animal Ethics Committee and conducted in accordance with the Australian Code for the Care and Use of Animals for Scientific Purposes. NSG‐A24 (NOD.Cg‐*Prkdc^scid^*IL2rg^tm1Whl^ Tg (HLA‐A24/H2‐D/B2M) 3Dvs/Sz) mice transgenic for human HLA‐A*2402^52^ were kindly provided by Professor Leonard Shultz (The Jackson Laboratory, Bar Harbor, ME, USA). Humanised NSG‐A24 mice were generated by irradiation (10 Gy) of 2‐ to 5‐day‐old neonates followed by intrahepatic injection of human HLA‐A*2402^+^ progenitor cells as described previously for NSG‐A2 mice.^49^ Reconstitution of human CD45^+^ cells was confirmed at 10–14 weeks, after which humanised mice received 2 × 50 µg subcutaneous (s.c.) injections of Flt3L (Bio‐X Cell, West Lebanon, NH, USA) separated by an interval of 4 days.

CD141^+^ DCs were flow‐purified from humanised mouse bone marrow suspensions using a FACSAria Fusion Sorter (BD Biosciences) after first depleting mouse CD45^+^ and human CD3^+^, CD14^+^, CD19^+^, CD20^+^ and CD34^+^ cells by magnetic separation (Dynabeads Magnetic Beads, Thermo Fisher Scientific), and then staining with Live/Dead Aqua (BioLegend), anti‐CD45‐APC‐Cy7 (clone H130, lineage‐specific Abs conjugated to Pacific Blue (see above), and anti‐HLA‐DR‐PE‐Cy7 (clone L243), anti‐CD1c‐PE (clone L161), anti‐CD123‐PerCP‐Cy5.5 (clone 6H6) and anti‐CD141‐APC (clone 1A4, BD Biosciences) as described previously.^49^

### T‐cell lines, peptides, and tetramers

T‐cell lines specific for the HLA‐A*2402‐restricted WT1_235–245_ epitope (CMTWNQMNL) were generated after transfer of a WT1_235–243_‐specific T‐cell receptor (TCR)‐encoding lentivirus into primary UCB CD8^+^ T cells (Supplementary figure 3). T‐cell lines specific for the HLA‐A*0201‐restricted WT1_126–134_ epitope (RMFPNAPYL) were generated from healthy donors by expansion of CD8^+^ T cells with WT1_126–134_ peptide‐pulsed moDCs, followed by enrichment using streptamer technology, single cell FACs sorting and expansion of clones as described previously.^72^ The corresponding peptides were purchased from GL Biochem (Shanghai, China). WT1_235–243_/HLA‐A*2402 tetramers were generated as described previously.^73^

### Cross‐presentation assays

Flow‐purified HLA‐A*2402^+^ or HLA‐A*0201^+^ CD141^+^ DCs generated from *in vitro* cultures or humanised mice were incubated with 10 µg mL^−1^ CLEC9A‐WT1, DEC‐205‐WT1, or control‐WT1 vaccines, or an equivalent amount of recombinant WT1 protein (0.7 µg mL^−1^, Mimotopes, Mulgrave, VIC, Australia) or no Ag, for 2 h at 37 °C in RPMI 1640 medium (Gibco Thermo Fisher Scientific) supplemented with 10% FBS, HEPES (10 mm), sodium pyruvate (1 mm), penicillin/streptomycin (100 U mL^−1^), GlutaMAX (2 mm) and non‐essential amino acids (0.1 mm) (all from Gibco Thermo Fisher Scientific), together with 2‐mercaptoethanol (50 µm, Sigma‐Aldrich). After washing, DCs were incubated with WT1‐specific T‐cell lines for 16–20 h at 37 °C on ELISPOT plates (Merck Millipore, Darmstadt, Germany) precoated with an anti‐human IFN‐γ Ab (clone 1‐D1K, Mabtech, Nacka Strand, Sweden). Wells were developed using anti‐human IFN‐γ‐biotin (clone 7‐B6‐1), streptavidin–alkaline phosphatase and BCIP/NBT‐plus substrate (all from Mabtech). Spots were counted using an AID ELISPOT reader (Autoimmun Diagnostika GmbH, Strassberg, Germany) and ImmunoSpot CTL (Cellular Technology Limited, Cleveland, OH, USA).

### CD8^+^ T‐cell priming assays

Human CD34^+^ HSCs were incubated overnight in X‐VIVO 15 medium (Lonza, Basel, Switzerland) supplemented with 10% FBS (Gibco Thermo Fisher Scientific), Flt3L (50 ng mL^−1^), SCF (50 ng mL^−1^), IL‐3 (20 ng mL^−1^) and TPO (50 ng mL^−1^) (all from PeproTech). Cells were then transferred to plates precoated with RetroNectin (20 µg mL^−1^, Clontech, Takara, Mountain View, CA, USA) and blocked with human serum albumin (2%). A WT1_235–243_‐specific TCR‐encoding lentivirus was added at a multiplicity of infection of 100, and HSCs were incubated for a further 24 h, washed and engrafted intrahepatically into NSG‐A24 mice. Reconstitution of human CD45^+^ cells was confirmed at 10–12 weeks, after which humanised mice received 2 × 50 µg s.c. injections of Flt3L (Bio‐X Cell) separated by an interval of 4 days. Spleens from humanised mice were digested in collagenase IV (Worthington Biochemical, Lakewood, NJ, USA) and DNase I (Roche, Basel, Switzerland), separated over a Percoll density gradient, and enriched for human leucocytes using a Mouse/Human Chimera EasySep Kit (Stemcell Technologies). Expression of the WT1_235–243_‐specific TCR was confirmed by staining with the corresponding tetramer conjugated to APC, followed by anti‐mouse CD45‐V500 (clone 30‐F11, BD Biosciences), anti‐rat CD2‐PE (clone OX‐34), and the anti‐human Abs anti‐CD45‐BUV395 (clone HI30, BD Biosciences), anti‐CD3‐Pacific Blue or anti‐CD3‐BV711 (clone OKT3), anti‐CD8‐PE‐Cy7 (clone RPA‐T8), anti‐CD197‐BV711 (clone 3D12, BD Biosciences) and anti‐CD45RA‐BV786 (clone HI100, BD Biosciences). Binding of CLEC9A‐WT1 and DEC‐205‐WT1 to humanised mouse splenocytes was confirmed by staining with the chimeric Abs, followed by anti‐human IgG4‐biotin (Invitrogen Thermo Fisher Scientific), and then SA‐PE (Invitrogen Thermo Fisher Scientific), Live/Dead Aqua (BioLegend), anti‐mouse CD45‐V500 (clone 30‐F11, BD Biosciences), and the anti‐human Abs anti‐CD45‐APC‐Cy7 (clone H130), anti‐CD3‐BV711 (clone OKT3), anti‐CD19‐Pacific Blue (clone HIB19), anti‐CD20‐Pacific Blue (clone 2H7), anti‐HLA‐DR‐PerCP‐Cy5.5 (clone L243), anti‐CD11c‐PE‐CF594 (clone B‐ly6, BD Biosciences), anti‐CD14‐APC (clone HCD14), anti‐CD16‐BV785 (clone 3G8), anti‐CD56‐FITC (clone HCD56), anti‐CD8‐BUV395 (clone RPA‐T8), anti‐CD1c‐Alexa Fluor 700 (clone L161), anti‐CD11b‐BV650 (clone M1/70), anti‐CD123‐BUV395 (clone 7G3, BD Biosciences) and anti‐CD141‐PE‐Cy7 (clone 1A4, BD Biosciences). Data were acquired using an LSRFortessa X‐20 (BD Biosciences) and analysed using FlowJo software version 9 or 10 (Tree Star Inc.).

For *ex vivo* ELISPOT assays, splenocytes enriched with human CD45^+^ cells were incubated overnight with 10 µg mL^−1^ CLEC9A‐WT1, DEC‐205‐WT1 or control‐WT1 vaccines. For CD8^+^ T‐cell priming assays, splenocytes enriched with human CD45^+^ cells were cultured with the same chimeric Abs, WT1_235–243_ peptide or no Ag in the presence of polyIC and R848 (InvivoGen, San Diego, CA, USA) for 2 h at 37 °C, washed and then cultured for 9–10 days in RPMI 1640 medium (Gibco Thermo Fisher Scientific) supplemented with IL‐2 (100 U mL^−1^, Invitrogen Thermo Fisher Scientific), IL‐7 (10 ng mL^−1^, Invitrogen Thermo Fisher Scientific) and IL‐15 (20 ng mL^−1^, PeproTech). Expansion of WT1_235–243_‐specific CD8^+^ T cells was analysed by staining with the corresponding tetramer conjugated to APC, followed by anti‐rat CD2‐PE (clone OX‐34) and the anti‐human Abs anti‐CD45‐BUV395 (clone HI30, BD Biosciences), anti‐CD3‐Pacific Blue or anti‐CD3‐BV711 (clone OKT3), and anti‐CD8‐PE‐Cy7 (clone RPA‐T8).

### Statistical analysis

Data were tested for normality using the Kolmogorov–Smirnoff test. Multigroup comparisons were performed using a repeated measures one‐way ANOVA or the non‐parametric equivalent (Friedman test), followed by the appropriate post‐test (Tukey or Dunn). Paired comparisons were performed using a paired *t*‐test or the non‐parametric Wilcoxon's signed rank test. Statistical significance was defined as a *P* value ≤ 0.05. All tests were performed using Prism version 5 (GraphPad, San Diego, CA, USA).

## Conflict of interest

The authors declare no conflict of interest.

## Author contribution

**Frances E Pearson:** Formal analysis; Investigation; Methodology; Project administration; Writing‐original draft; Writing‐review & editing. **Kirsteen M Tullett:** Investigation; Methodology; Writing‐review & editing. **Ingrid M Leal‐Rojas** **Ingrid M Leal‐Rojas:** Data curation; Formal analysis; Investigation; Methodology; Writing‐original draft; Writing‐review & editing. **Oscar L Haigh:** Investigation; Methodology; Writing‐review & editing. **Kelly‐Anne Masterman:** Data curation; Formal analysis; Investigation; Methodology; Writing‐original draft; Writing‐review & editing. **Carina Walpole:** Investigation; Methodology; Writing‐review & editing. **John S Bridgeman:** Methodology; Resources; Writing‐review & editing. **James E McLaren:** Investigation; Methodology; Resources; Writing‐review & editing. **Kristin Ladell:** Investigation; Methodology; Resources. **Kelly Miners:** Investigation; Methodology; Resources. **Sian Llewellyn‐Lacey:** Investigation; Methodology; Resources. **David A Price:** Funding acquisition; Investigation; Methodology; Resources; Writing‐review & editing. **Antje Tunger:** Investigation; Methodology; Resources. **Marc Schmitz:** Investigation; Methodology; Resources; Writing‐review & editing. **John J Miles:** Investigation; Methodology; Writing‐review & editing. **Mireille H Lahoud:** Conceptualization; Formal analysis; Methodology; Writing‐review & editing. **Kristen J Radford:** Conceptualization; Data curation; Formal analysis; Funding acquisition; Investigation; Project administration; Supervision; Writing‐original draft; Writing‐review & editing.

## Supporting information

Supplementary figures 1‐4

## References

[1] O'Brien LJ , Guillerey C , Radford KJ . Can dendritic cell vaccination prevent leukemia relapse? Cancers (Basel) 2019; 11: 875. 10.3390/cancers11060875 PMC6627518 31234526

[2] Anguille S , Smits EL , Lion E , van Tendeloo VF , Berneman ZN . Clinical use of dendritic cells for cancer therapy. Lancet Oncol 2014; 15: e257–e267. 24872109 10.1016/S1470-2045(13)70585-0

[3] Radford KJ , Tullett KM , Lahoud MH . Dendritic cells and cancer immunotherapy. Curr Opin Immunol 2014; 27: 26–32. 24513968 10.1016/j.coi.2014.01.005

[4] O'Keeffe M , Mok WH , Radford KJ . Human dendritic cell subsets and function in health and disease. Cell Mol Life Sci 2015; 72: 4309–4325. 26243730 10.1007/s00018-015-2005-0 PMC11113503

[5] Van Acker HH , Versteven M , Lichtenegger FS *et al* Dendritic cell‐based immunotherapy of acute myeloid leukemia. J Clin Med 2019; 8: 579. 10.3390/jcm8050579 PMC6572115 31035598

[6] Cheever MA , Allison JP , Ferris AS *et al* The prioritization of cancer antigens: a national cancer institute pilot project for the acceleration of translational research. Clin Cancer Res 2009; 15: 5323–5337. 19723653 10.1158/1078-0432.CCR-09-0737 PMC5779623

[7] Oka Y , Tsuboi A , Oji Y , Kawase I , Sugiyama H . WT1 peptide vaccine for the treatment of cancer. Curr Opin Immunol 2008; 20: 211–220. 18502632 10.1016/j.coi.2008.04.009

[8] Cilloni D , Renneville A , Hermitte F *et al* Real‐time quantitative polymerase chain reaction detection of minimal residual disease by standardized WT1 assay to enhance risk stratification in acute myeloid leukemia: a European LeukemiaNet study. J Clin Oncol 2009; 27: 5195–5201. 19752335 10.1200/JCO.2009.22.4865

[9] Nakata J , Oji Y , Oka Y , Sugiyama H . What should we tackle next in acute myeloid leukemia? Wilms tumor gene 1 vaccine therapy would be a promising and versatile strategy for acute myeloid leukemia. Expert Rev Hematol 2019; 12: 211–213. 30882253 10.1080/17474086.2019.1593824

[10] Oka Y , Tsuboi A , Nakata J *et al* Wilms' tumor gene 1 (WT1) peptide vaccine therapy for hematological malignancies: from CTL epitope identification to recent progress in clinical studies including a cure‐oriented strategy. Oncol Res Treat 2017; 40: 682–690. 29041012 10.1159/000481353

[11] Nakata J , Nakae Y , Kawakami M *et al* Wilms tumour 1 peptide vaccine as a cure‐oriented post‐chemotherapy strategy for patients with acute myeloid leukaemia at high risk of relapse. Br J Haematol 2018; 182: 287–290. 28542830 10.1111/bjh.14768

[12] Rezvani K , Yong AS , Mielke S *et al* Repeated PR1 and WT1 peptide vaccination in Montanide‐adjuvant fails to induce sustained high‐avidity, epitope‐specific CD8+ T cells in myeloid malignancies. Haematologica 2011; 96: 432–440. 21134985 10.3324/haematol.2010.031674 PMC3046275

[13] Kuball J , de Boer K , Wagner E *et al* Pitfalls of vaccinations with WT1‐, Proteinase3‐ and MUC1‐derived peptides in combination with MontanideISA51 and CpG7909. Cancer Immunol Immunother 2011; 60: 161–171. 20963411 10.1007/s00262-010-0929-7 PMC3024516

[14] Anguille S , Van de Velde AL , Smits EL *et al* Dendritic cell vaccination as postremission treatment to prevent or delay relapse in acute myeloid leukemia. Blood 2017; 130: 1713–1721. 28830889 10.1182/blood-2017-04-780155 PMC5649080

[15] Van Tendeloo VF , Van de Velde A , Van Driessche A *et al* Induction of complete and molecular remissions in acute myeloid leukemia by Wilms' tumor 1 antigen‐targeted dendritic cell vaccination. Proc Natl Acad Sci USA 2010; 107: 13824–13829. 20631300 10.1073/pnas.1008051107 PMC2922237

[16] Tullett KM , Lahoud MH , Radford KJ . Harnessing human cross‐presenting CLEC9A^+^XCR1^+^ dendritic cells for immunotherapy. Front Immunol 2014; 5: 239. 24904587 10.3389/fimmu.2014.00239 PMC4033245

[17] Bonifaz LC , Bonnyay DP , Charalambous A *et al* *In vivo* targeting of antigens to maturing dendritic cells via the DEC‐205 receptor improves T cell vaccination. J Exp Med 2004; 199: 815–824. 15024047 10.1084/jem.20032220 PMC2212731

[18] Johnson TS , Mahnke K , Storn V *et al* Inhibition of melanoma growth by targeting of antigen to dendritic cells via an anti‐DEC‐205 single‐chain fragment variable molecule. Clin Cancer Res 2008; 14: 8169–8177. 19088032 10.1158/1078-0432.CCR-08-1474

[19] Mahnke K , Qian Y , Fondel S , Brueck J , Becker C , Enk AH . Targeting of antigens to activated dendritic cells *in vivo* cures metastatic melanoma in mice. Cancer Res 2005; 65: 7007–7012. 16061687 10.1158/0008-5472.CAN-05-0938

[20] Dhodapkar MV , Sznol M , Zhao B *et al* Induction of antigen‐specific immunity with a vaccine targeting NY‐ESO‐1 to the dendritic cell receptor DEC‐205. Sci Transl Med 2014; 6: 232ra51. 10.1126/scitranslmed.3008068 PMC6151129 24739759

[21] Griffiths EA , Srivastava P , Matsuzaki J *et al* NY‐ESO‐1 vaccination in combination with decitabine induces antigen‐specific T‐lymphocyte responses in patients with Myelodysplastic syndrome. Clin Cancer Res 2018; 24: 1019–1029. 28947565 10.1158/1078-0432.CCR-17-1792 PMC5844797

[22] Odunsi K , Matsuzaki J , James SR *et al* Epigenetic potentiation of NY‐ESO‐1 vaccine therapy in human ovarian cancer. Cancer Immunol Res 2014; 2: 37–49. 24535937 10.1158/2326-6066.CIR-13-0126 PMC3925074

[23] Kato M , McDonald KJ , Khan S *et al* Expression of human DEC‐205 (CD205) multilectin receptor on leukocytes. Int Immunol 2006; 18: 857–869. 16581822 10.1093/intimm/dxl022

[24] Bottcher JP , Reis ESC . The role of type 1 conventional dendritic cells in cancer immunity. Trends Cancer 2018; 4: 784–869. 30352680 10.1016/j.trecan.2018.09.001 PMC6207145

[25] Sanchez‐Paulete AR , Teijeira A , Cueto FJ *et al* Antigen cross‐presentation and T‐cell cross‐priming in cancer immunology and immunotherapy. Ann Oncol 2017; 28: xii44–xii55. 28945841 10.1093/annonc/mdx237

[26] Cancel JC , Crozat K , Dalod M , Mattiuz R . Are conventional type 1 dendritic cells critical for protective antitumor immunity and how? Front Immunol 2019; 10: 9. 30809220 10.3389/fimmu.2019.00009 PMC6379659

[27] Kline DE , MacNabb BW , Chen X , Chan WC , Fosco D , Kline J . CD8α^+^ dendritic cells dictate leukemia‐specific CD8^+^ T cell fates. J Immunol 2018; 201: 3759–3769. 30420437 10.4049/jimmunol.1801184 PMC6444187

[28] Broz ML , Binnewies M , Boldajipour B *et al* Dissecting the tumor myeloid compartment reveals rare activating antigen‐presenting cells critical for T cell immunity. Cancer Cell 2014; 26: 638–652. 25446897 10.1016/j.ccell.2014.09.007 PMC4254577

[29] Spranger S , Dai D , Horton B , Gajewski TF . Tumor‐residing Batf3 dendritic cells are required for effector T cell trafficking and adoptive T cell therapy. Cancer Cell 2017; 31: 711–723.e4. 28486109 10.1016/j.ccell.2017.04.003 PMC5650691

[30] Bommareddy PK , Aspromonte S , Zloza A , Rabkin SD , Kaufman HL . MEK inhibition enhances oncolytic virus immunotherapy through increased tumor cell killing and T cell activation. Sci Transl Med 2018; 10: eaau0417. 30541787 10.1126/scitranslmed.aau0417 PMC7593827

[31] Dai P , Wang W , Yang N *et al* Intratumoral delivery of inactivated modified vaccinia virus Ankara (iMVA) induces systemic antitumor immunity via STING and Batf3‐dependent dendritic cells. Sci Immunol 2017; 2: eaal1713. 28763795 10.1126/sciimmunol.aal1713 PMC5559204

[32] Sanchez‐Paulete AR , Cueto FJ , Martinez‐Lopez M *et al* Cancer immunotherapy with immunomodulatory anti‐CD137 and anti‐PD‐1 monoclonal antibodies requires BATF3‐dependent dendritic cells. Cancer Discov 2016; 6: 71–79. 26493961 10.1158/2159-8290.CD-15-0510 PMC5036540

[33] Salmon H , Idoyaga J , Rahman A *et al* Expansion and activation of CD103^+^ dendritic cell progenitors at the tumor site enhances tumor responses to therapeutic PD‐L1 and BRAF inhibition. Immunity 2016; 44: 924–938. 27096321 10.1016/j.immuni.2016.03.012 PMC4980762

[34] Jongbloed SL , Kassianos AJ , McDonald KJ *et al* Human CD141^+^ (BDCA‐3)^+^ dendritic cells (DCs) represent a unique myeloid DC subset that cross‐presents necrotic cell antigens. J Exp Med 2010; 207: 1247–1260. 20479116 10.1084/jem.20092140 PMC2882828

[35] Bachem A , Guttler S , Hartung E *et al* Superior antigen cross‐presentation and XCR1 expression define human CD11c^+^CD141^+^ cells as homologues of mouse CD8^+^ dendritic cells. J Exp Med 2010; 207: 1273–1281. 20479115 10.1084/jem.20100348 PMC2882837

[36] Crozat K , Guiton R , Contreras V *et al* The XC chemokine receptor 1 is a conserved selective marker of mammalian cells homologous to mouse CD8α^+^ dendritic cells. J Exp Med 2010; 207: 1283–1292. 20479118 10.1084/jem.20100223 PMC2882835

[37] Haniffa M , Shin A , Bigley V *et al* Human tissues contain CD141^hi^ cross‐presenting dendritic cells with functional homology to mouse CD103^+^ nonlymphoid dendritic cells. Immunity 2012; 37: 60–73. 22795876 10.1016/j.immuni.2012.04.012 PMC3476529

[38] Chiang MC , Tullett KM , Lee YS *et al* Differential uptake and cross‐presentation of soluble and necrotic cell antigen by human DC subsets. Eur J Immunol 2016; 46: 329–339. 26542182 10.1002/eji.201546023

[39] Barry KC , Hsu J , Broz ML *et al* A natural killer‐dendritic cell axis defines checkpoint therapy‐responsive tumor microenvironments. Nat Med 2018; 24: 1178–1191. 29942093 10.1038/s41591-018-0085-8 PMC6475503

[40] Bottcher JP , Bonavita E , Chakravarty P *et al* NK cells stimulate recruitment of cDC1 into the tumor microenvironment promoting cancer immune control. Cell 2018; 172: 1022–1037.e14. 29429633 10.1016/j.cell.2018.01.004 PMC5847168

[41] Caminschi I , Proietto AI , Ahmet F *et al* The dendritic cell subtype‐restricted C‐type lectin Clec9A is a target for vaccine enhancement. Blood 2008; 112: 3264–3273. 18669894 10.1182/blood-2008-05-155176 PMC2569177

[42] Sancho D , Mourao‐Sa D , Joffre OP *et al* Tumor therapy in mice via antigen targeting to a novel, DC‐restricted C‐type lectin. J Clin Invest 2008; 118: 2098–2110. 18497879 10.1172/JCI34584 PMC2391066

[43] Ahrens S , Zelenay S , Sancho D *et al* F‐actin is an evolutionarily conserved damage‐associated molecular pattern recognized by DNGR‐1, a receptor for dead cells. Immunity 2012; 36: 635–645. 22483800 10.1016/j.immuni.2012.03.008

[44] Zhang JG , Czabotar PE , Policheni AN *et al* The dendritic cell receptor Clec9A binds damaged cells via exposed actin filaments. Immunity 2012; 36: 646–657. 22483802 10.1016/j.immuni.2012.03.009

[45] Sancho D , Joffre OP , Keller AM *et al* Identification of a dendritic cell receptor that couples sensing of necrosis to immunity. Nature 2009; 458: 899–903. 19219027 10.1038/nature07750 PMC2671489

[46] Idoyaga J , Lubkin A , Fiorese C *et al* Comparable T helper 1 (Th1) and CD8 T‐cell immunity by targeting HIV gag p24 to CD8 dendritic cells within antibodies to Langerin, DEC205, and Clec9A. Proc Natl Acad Sci USA 2011; 108: 2384–2389. 21262813 10.1073/pnas.1019547108 PMC3038758

[47] Tullett KM , Leal Rojas IM , Minoda Y *et al* Targeting CLEC9A delivers antigen to human CD141^+^ DC for CD4^+^ and CD8^+^T cell recognition. JCI Insight 2016; 1: e87102. 27699265 10.1172/jci.insight.87102 PMC5033826

[48] Balan S , Ollion V , Colletti N *et al* Human XCR1^+^ dendritic cells derived *in vitro* from CD34^+^ progenitors closely resemble blood dendritic cells, including their adjuvant responsiveness, contrary to monocyte‐derived dendritic cells. J Immunol 2014; 193: 1622–1635. 25009205 10.4049/jimmunol.1401243 PMC4120898

[49] Minoda Y , Virshup I , Leal Rojas I *et al* Human CD141^+^ dendritic cell and CD1c^+^ dendritic cell undergo concordant early genetic programming after activation in humanized mice in vivo. Front Immunol 2017; 8: 1419. 29163495 10.3389/fimmu.2017.01419 PMC5670352

[50] Pearson FE , Chang K , Minoda Y *et al* Activation of human CD141^+^ and CD1c^+^ dendritic cells in vivo with combined TLR3 and TLR7/8 ligation. Immunol Cell Biol 2018; 96: 390–400. 29344995 10.1111/imcb.12009

[51] Ding Y , Wilkinson A , Idris A *et al* FLT3‐ligand treatment of humanized mice results in the generation of large numbers of CD141^+^ and CD1c^+^ dendritic cells *in vivo* . J Immunol 2014; 192: 1982–1989. 24453245 10.4049/jimmunol.1302391

[52] Najima Y , Tomizawa‐Murasawa M , Saito Y *et al* Induction of WT1‐specific human CD8^+^ T cells from human HSCs in HLA class I Tg NOD/SCID/IL2rgKO mice. Blood 2016; 127: 722–734. 26702062 10.1182/blood-2014-10-604777 PMC4751022

[53] Giannoni F , Hardee CL , Wherley J *et al* Allelic exclusion and peripheral reconstitution by TCR transgenic T cells arising from transduced human hematopoietic stem/progenitor cells. Mol Ther 2013; 21: 1044–1054. 23380815 10.1038/mt.2013.8 PMC3666644

[54] Zelenay S , Keller AM , Whitney PG *et al* The dendritic cell receptor DNGR‐1 controls endocytic handling of necrotic cell antigens to favor cross‐priming of CTLs in virus‐infected mice. J Clin Invest 2012; 122: 1615–1627. 22505458 10.1172/JCI60644 PMC3336984

[55] Alcantara‐Hernandez M , Leylek R , Wagar LE *et al* High‐dimensional phenotypic mapping of human dendritic cells reveals interindividual variation and tissue specialization. Immunity 2017; 47: 1037–1050.e6. 29221729 10.1016/j.immuni.2017.11.001 PMC5738280

[56] Hsu JL , Bryant CE , Papadimitrious MS *et al* A blood dendritic cell vaccine for acute myeloid leukemia expands anti‐tumor T cell responses at remission. Oncoimmunology 2018; 7: e1419114. 29632738 10.1080/2162402X.2017.1419114 PMC5889209

[57] Sawada A , Inoue M , Kondo O *et al* Feasibility of cancer immunotherapy with WT1 peptide vaccination for solid and hematological malignancies in children. Pediatr Blood Cancer 2016; 63: 234–241. 26469989 10.1002/pbc.25792

[58] Hashii Y , Sato E , Ohta H , Oka Y , Sugiyama H , Ozono K . WT1 peptide immunotherapy for cancer in children and young adults. Pediatr Blood Cancer 2010; 55: 352–355. 20582983 10.1002/pbc.22522

[59] Hashii Y , Sato‐Miyashita E , Matsumura R *et al* WT1 peptide vaccination following allogeneic stem cell transplantation in pediatric leukemic patients with high risk for relapse: successful maintenance of durable remission. Leukemia 2012; 26: 530–532. 21869838 10.1038/leu.2011.226

[60] Higgins M , Curigliano G , Dieras V *et al* Safety and immunogenicity of neoadjuvant treatment using WT1‐immunotherapeutic in combination with standard therapy in patients with WT1‐positive Stage II/III breast cancer: a randomized Phase I study. Breast Cancer Res Treat 2017; 162: 479–488. 28176175 10.1007/s10549-017-4130-y PMC5332485

[61] Nishida S , Ishikawa T , Egawa S *et al* Combination gemcitabine and WT1 peptide vaccination improves progression‐free survival in advanced pancreatic ductal adenocarcinoma: a phase II randomized study. Cancer Immunol Res 2018; 6: 320–331. 29358173 10.1158/2326-6066.CIR-17-0386

[62] Yanagisawa R , Koizumi T , Koya T *et al* WT1‐pulsed dendritic cell vaccine combined with chemotherapy for resected pancreatic cancer in a phase I study. Anticancer Res 2018; 38: 2217–2225. 29599342 10.21873/anticanres.12464

[63] Tsuboi A , Hashimoto N , Fujiki F *et al* A phase I clinical study of a cocktail vaccine of Wilms' tumor 1 (WT1) HLA class I and II peptides for recurrent malignant glioma. Cancer Immunol Immunother 2019; 68: 331–340. 30430205 10.1007/s00262-018-2274-1 PMC6394509

[64] Anandasabapathy N , Breton G , Hurley A *et al* Efficacy and safety of CDX‐301, recombinant human Flt3L, at expanding dendritic cells and hematopoietic stem cells in healthy human volunteers. Bone Marrow Transplant 2015; 50: 924–930. 25915810 10.1038/bmt.2015.74 PMC4532305

[65] Deng L , Liang H , Xu M *et al* STING‐dependent cytosolic DNA sensing promotes radiation‐induced type I interferon‐dependent antitumor immunity in immunogenic tumors. Immunity 2014; 41: 843–852. 25517616 10.1016/j.immuni.2014.10.019 PMC5155593

[66] Vanpouille‐Box C , Alard A , Aryankalayil MJ *et al* DNA exonuclease Trex1 regulates radiotherapy‐induced tumour immunogenicity. Nat Commun 2017; 8: 15618. 28598415 10.1038/ncomms15618 PMC5472757

[67] Cohn L , Chatterjee B , Esselborn F *et al* Antigen delivery to early endosomes eliminates the superiority of human blood BDCA3^+^ dendritic cells at cross presentation. J Exp Med 2013; 210: 1049–1063. 23569326 10.1084/jem.20121251 PMC3646496

[68] Graham JP , Authie P , Yu CI *et al* Targeting dendritic cells in humanized mice receiving adoptive T cells via monoclonal antibodies fused to Flu epitopes. Vaccine 2016; 34: 4857–4865. 27595442 10.1016/j.vaccine.2016.08.071 PMC5598757

[69] Binnewies M , Mujal AM , Pollack JL *et al* Unleashing type‐2 dendritic cells to drive protective antitumor CD4^+^ T cell immunity. Cell 2019; 177: 556–571.e16. 30955881 10.1016/j.cell.2019.02.005 PMC6954108

[70] Li J , Ahmet F , Sullivan LC *et al* Antibodies targeting Clec9A promote strong humoral immunity without adjuvant in mice and non‐human primates. Eur J Immunol 2015; 45: 854–864. 25487143 10.1002/eji.201445127

[71] Law SC , Haigh OL , Walpole CM *et al* Simple, rapid and inexpensive typing of common HLA class I alleles for immunological studies. J Immunol Methods 2019; 465: 72–76. 30537479 10.1016/j.jim.2018.12.002

[72] Tunger A , Wehner R , von Bonin M *et al* Generation of high‐avidity, WT1‐reactive CD8^+^ cytotoxic T cell clones with anti‐leukemic activity by streptamer technology. Leuk Lymphoma 2017; 58: 1246–1249. 27852136 10.1080/10428194.2016.1233538

[73] Price DA , Brenchley JM , Ruff LE *et al* Avidity for antigen shapes clonal dominance in CD8^+^ T cell populations specific for persistent DNA viruses. J Exp Med 2005; 202: 1349–1361. 16287711 10.1084/jem.20051357 PMC2212993

