# Supplementary material for: Human CLEC9A antibodies deliver Wilms' tumor 1 (WT1) antigen to CD141+ dendritic cells to activate naïve and memory WT1‐specific CD8+ T cells
Source: Clin Transl Immunology. 2020 Jun 12;9(6):e1141. doi: 10.1002/cti2.1141 (PMC7292901; doi:10.1002/cti2.1141)
Supplement: Supplementary file 1 — Supplementary figures 1‐4 [file CTI2-9-e1141-s001.pdf]

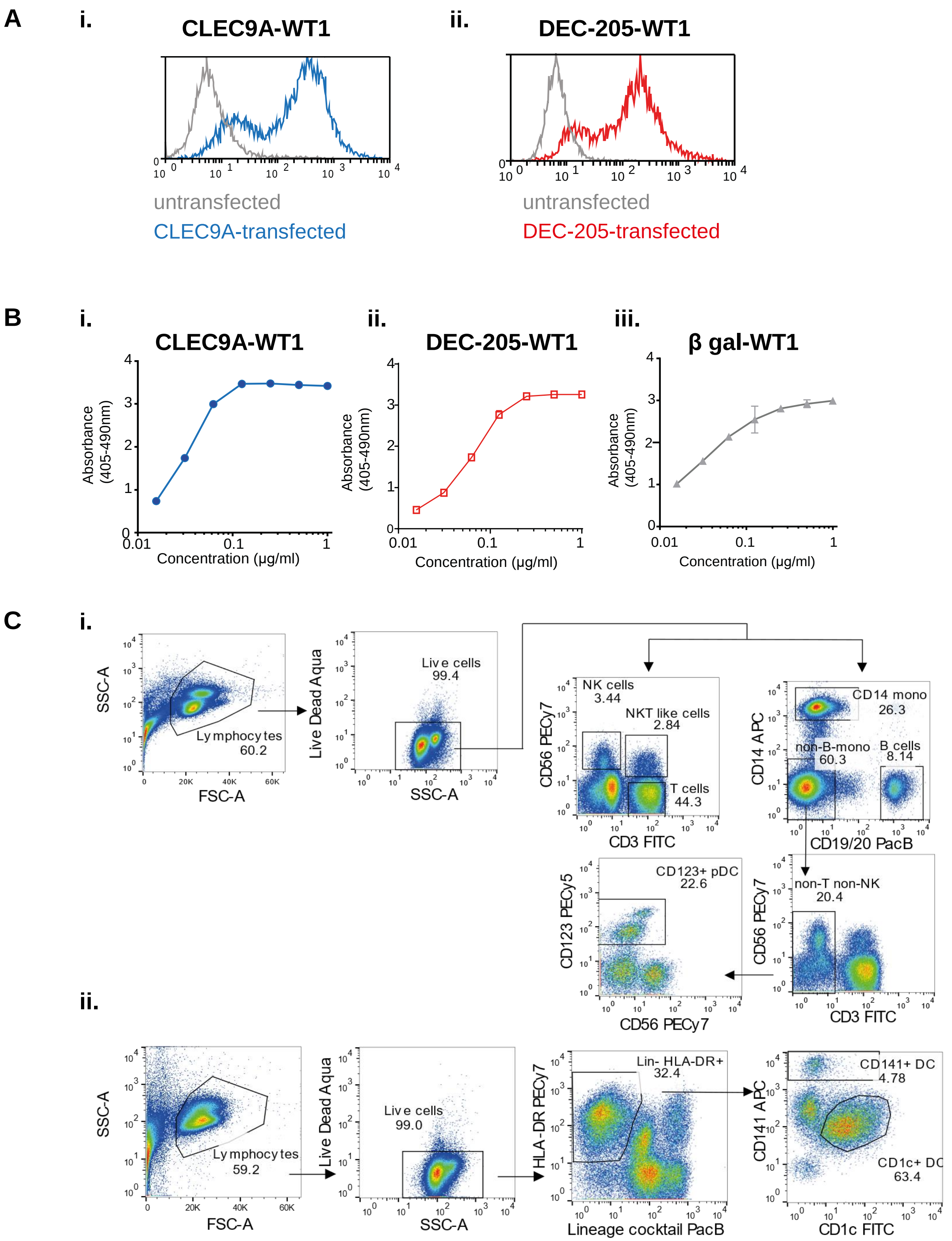

Supplementary figure 1 (Related to manuscript Figure 1): Validation of chimeric CLEC9A and DEC-205 and β-gal (control) Ab specificity. **A.** Binding of CLEC9A-WT1 (i) or DEC-205-WT1 (ii) chimeric Abs to mammalian 293F cells transiently transfected with full-length CLEC9A (blue), DEC-205 (red) or untransfected (grey). Chimeric Ab binding was detected using anti-human IgG4-biotin (Life technologies) and streptavidin-PE (BD Pharmingen) by flow cytometry. **B.** Binding of CLEC9A-WT1 (i), DEC205-WT1 (ii) and β-gal (control)-WT1 (iii) to respective CLEC9A, DEC-205 or β-gal recombinant proteins or peptides by ELISA, detected using anti-human IgG4-biotin and streptavidin-HRP (GE Healthcare) and visualised with ABTS as previously described (Tullett et al, JCI Insight. 2016 May 19;1(7):e87102. doi: 10.1172/jci.insight.87102). **C.** Gating strategy for identification of immune cell subsets from (i) PBMC (ii) mDC-enriched PBMC from healthy peripheral blood for validation of chimeric Ab binding.

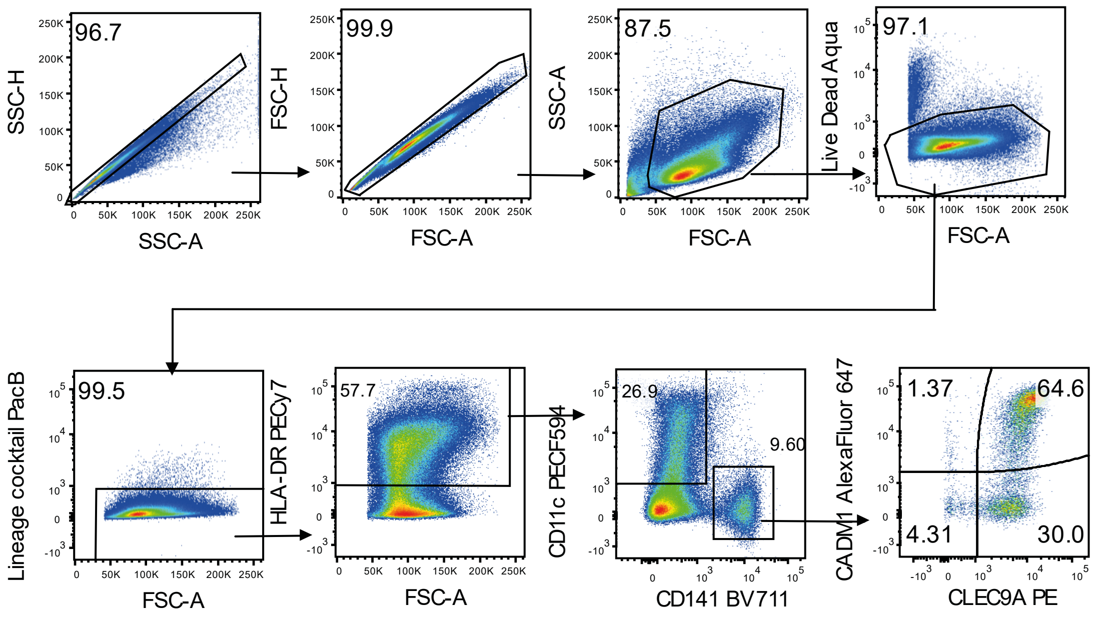

Supplementary figure 2 (Related to manuscript Figure 2): Generation and isolation of CD141$^+$ DC from CD34+ HSC in vitro. A. CD141$^+$ DC were identified and sorted as live, singlet cells that were lineage cocktail (CD2, CD14, CD16, CD19, CD20)$^-$, HLA-DR$^+$, CD11c^{low} and CD141$^+$. The majority of the CD141+ cells expressed CD141+ DC-specific markers CLEC9A and CADM1.

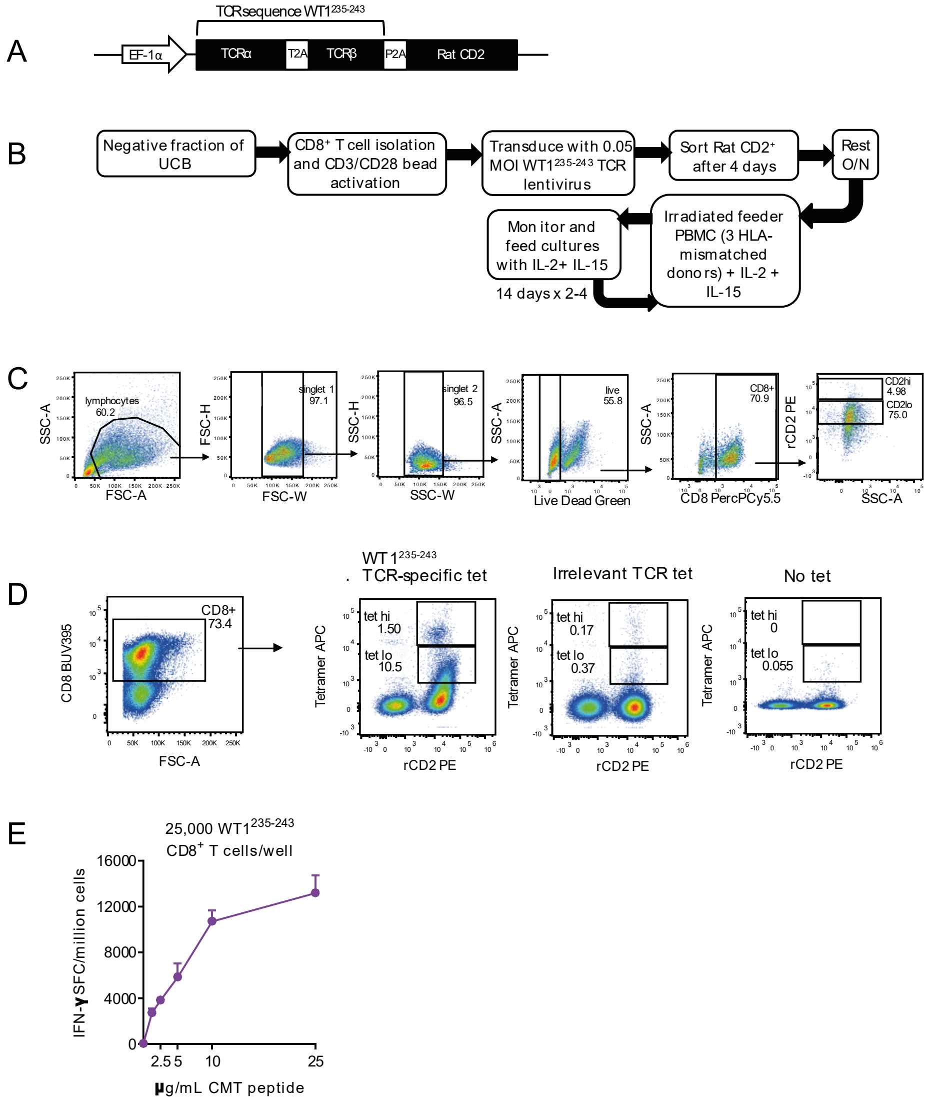

Supplementary figure 3: Generation and validation of a CD8⁺ T cell line specific for WT1$_{235-243}$. CD8$^+$ T cells expressing a TCR specific for WT1$_{235-243}$ were generated using a third generation lentiviral system. **A.** Lentiviral expression cassette encoding the TCR-α and β genes of the HLA-A*2402-restricted WT1$_{235-243}$ specific CTL clone TAK-1 (Ohminami H et al Blood 2000 Jan 1;95(1):286-93) and the rat CD2 reporter gene under control of mammalian Elongation Factor-1α promoter in the pELN vector. T2A and P2A cleavage sequences allow separate co-expression of TCR-α, -β and rat CD2. Lentiviral particles were generated by co-transfection of HEK 293T cells with the pELN transfer vector and packaging plasmids pVSVg (envelope), pMDLg/pRRE (gag/pol) and pRSV.rev (rev) in Lipofectamine 2000. Virus was concentrated by ultracentrifugation from supernatants harvested 24-72 hr after infection.

B. Schematic of the generation of WT1$_{235-243}$ T cell lines. CD8$^+$ T cells were isolated from cord blood by negative selection using an EasySep Human CD8$^+$ T cell isolation kit (Stemcell) and cultured overnight with anti-CD3/CD28 Dynabeads (ThermoFisher), prior to infection with the WT1$_{235-243}$ TCR encoding lentivirus in the presence of Polybrene (Sigma) and 200U/mL IL-2. Rat CD2^{hi} CD8$^+$ T cells were sorted by FACS four days later using the gating strategy shown in **C** and rested overnight**.** CD2^{hi} sorted cells were expanded with 2-4 x 14 day cycles of stimulation with HLA-mismatched irradiated PBMC from 3 separate donors, 1 µg/mL phytohaeagglutinin (PHA, Remel, ThermoFisher), 25 ng/mL IL-15 and 200IU/mL IL-2. Fresh media and cytokines were replenished every 2-3 days **D.** Expression of rat CD2 and the WT1$_{235-243}$ TCR on the surface of the expanded CD8$^+$ T cell line using a WT1$_{235-243}$ – specific tetramer. **E.** Specificity of the WT1$_{235-243}$-specific CD8$^+$ TCR line confirmed by IFNγ production following stimulation with titrating concentrations of cognate peptide (CMTWNQMNL) by ELISPOT.

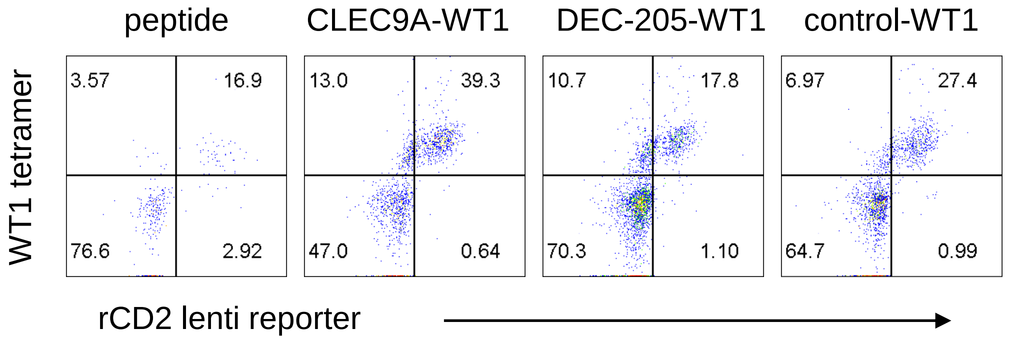

Supplementary figure 4 (Related to manuscript Figure 4). Representative dot plots showing expansion of $CD2^{+}$ $WT1_{235–243}$-specific TCR^{+} CD8+ T cells (gated on the CD3+ CD8+ T cell population) following expansion of humanized mouse splenocytes 8 days after incubation with cognate peptide or chimeric antibodies in the presence of poly I:C and R848.
